# Evaluating the Efficacy of Probiotics on Disease Progression, Quality of Life, and Nutritional Status Among Patients with Crohn’s Disease: A Multicenter, Randomized, Single-Blinded Controlled Trial

**DOI:** 10.3390/nu17040708

**Published:** 2025-02-17

**Authors:** Maha Hoteit, Mohamad Hellani, Mohamad Karaja, Nadeen Zayour, Zahra Sadek, Bilal Hotayt, Mahmoud Hallal

**Affiliations:** 1 Food Sciences Unit, National Council for Scientific Research of Lebanon (CNRS-L), Beirut P.O. Box 11-8281, Lebanon; maha.hoteit@cnrs.edu.lb; 2 PHENOL Research Program, Faculty of Public Health, Section 1, Lebanese University, Beirut P.O. Box 6573, Lebanon; nadeenzayour6@gmail.com (N.Z.); zahrasadek81@hotmail.com (Z.S.); 3 Organized Research Unit, Zahraa University Medical Center (ZHUMC), Beirut P.O. Box 90-361, Lebanon; 4 Gastroenterology Department, Faculty of Medical Science, Lebanese University, Beirut P.O. Box 14-6573, Lebanon; mhmad.hellani@hotmail.com (M.H.); samirkaraja01@outlook.com (M.K.); 5 Gastroenterology and Hepatology Department, Zahraa University Medical Center (ZHUMC), Beirut P.O. Box 90-361, Lebanon; 6 Physiotherapy Department, Faculty of Public Health, Islamic University of Lebanon, Khaldeh P.O. Box 30014, Lebanon; 7 Physiotherapy Department, Faculty of Public Health, Lebanese University, Beirut P.O. Box 6573-1, Lebanon; 8 Badaro Endoscopic Center, Moarbes Hospital, Beirut P.O. Box 50-223, Lebanon

**Keywords:** Crohn’s disease, randomized clinical trial, probiotics, *Lactobacillus*, *Bifidobacteria*, Lebanon

## Abstract

Background: There is growing interest in the role of gut microbiota in the pathophysiology of inflammatory bowel diseases (IBDs), including Crohn’s disease (CD). Probiotics have been proposed as a potential adjunct therapy for these conditions by altering the intestinal environment, although studies on their effectiveness have yielded mixed results. Aim: This study aims to evaluate the short-term (2 months) effects of a dietary supplement containing *Lactobacilli*, *Bifidobacteria*, and *Lactococcus bacillus* on disease progression, remission, quality of life, and nutritional intake in Lebanese patients with CD. Method: A multicenter, randomized, single-blind controlled trial was conducted in 2 medical centers in Beirut from 1 April 2024 to 1 August 2024. Recruitment, prescreening, screening, enrollment, and protocol implementation were carried out at both centers. Data were collected from 21 patients with CD, who were randomly assigned to the control group (n = 10) and the intervention group (n = 11). At baseline and after two months, participants underwent clinical assessments, WHOQOL-BREF evaluation, and 24 h dietary recalls. Follow-up visits included surveys on disease progression, quality of life, adherence, and adverse events, along with repeat body composition and anthropometric measurements. Results: Probiotic supplementation over two months did not significantly alter symptoms, flares, or hospitalizations outcomes between the control and intervention groups. However, the intervention group experienced notable increases in body weight (*p* = 0.01), BMI (*p* = 0.01), body fat mass (*p* = 0.04), and arm muscle circumference (*p* = 0.01). Nutrient intake patterns differed, with the intervention group showing increased consumption of calcium, riboflavin, and folate compared to controls (*p* = 0.01, *p* = 0.04, *p* = 0.013, respectively). Probiotic supplementation led to significant within-group increases in dietary fiber (*p* = 0.01), total sugar (*p* = 0.02), and caffeine (*p* = 0.01) among the intervention participants. Adverse effects in the intervention group were mild, including nausea (18.2%) and abdominal discomfort (9.1%). QOL improved significantly in the intervention group, particularly in physical (*p* = 0.03), psychological (*p* = 0.04), and environmental domains (*p* = 0.003), while the control group exhibited improvements only in psychological health. Conclusions: Overall, the findings suggest that probiotics can enhance body composition, nutrient intake, and certain aspects of QOL among CD patients, despite minimal impact on disease symptoms or dietary patterns.

## 1. Introduction

Recent progress in understanding the pathogenesis of inflammatory bowel disease (IBD) underscores the intricate relationship between genetic and environmental influences [1]. Environmental factors, such as the microbiome and dietary habits, play a pivotal role in shaping the epigenome, particularly during early-life windows of vulnerability, which may increase disease susceptibility [1]. Supporting this notion, a case–control study analyzing the interplay between the host genome, gut microbiota, and clinical phenotypes in 313 IBD patients and 582 healthy controls revealed significant microbiota alterations in healthy individuals with a high genetic risk for IBD [2]. Furthermore, the study highlighted variations in microbiota diversity linked to disease location, with lower diversity observed in ileal Crohn’s disease. Crohn’s disease (CD) is a chronic IBD characterized by recurring episodes of gastrointestinal inflammation, which significantly impact patients’ quality of life [3]. The exact etiology of CD remains elusive, but it is widely accepted that genetic susceptibility, environmental factors, and gut microbiota dysbiosis play critical roles in its pathogenesis [3]. Gut microbiota imbalances, particularly reduced levels of beneficial bacteria such as *Lactobacilli*, *Bifidobacteria*, and *Lactococcus*, are associated with impaired intestinal barrier function and dysregulated immune responses in CD [3,4]. Probiotic supplementation is nowadays emerging as a promising adjunctive therapy to restore microbial equilibrium, enhance gut barrier integrity, and mitigate inflammatory processes in CD [3,4,5]. Several clinical trials have evaluated the effects of probiotics on CD, with varying outcomes. For example, a randomized controlled trial by Fujimori et al. (2007) demonstrated that *Bifidobacterium breve* and *Lactobacillus casei* supplementation significantly improved clinical remission rates in CD patients compared to placebo [6]. One study investigated the effects of kefir consumption on fecal microflora and symptoms in 45 IBD patients, with 400 mL/day of kefir for 4 weeks. The results showed an increased *Lactobacillus* bacterial load and significant improvements in CD patients, including reductions in inflammation markers and increased hemoglobin levels. The authors concluded that kefir may modulate gut microbiota and improve the quality of life for CD patients in the short term [7]. A randomized, double-blind, placebo-controlled trial investigated the effects of a synbiotic (*Bifidobacterium longum* and Synergy 1) on 35 patients with active CD. Significant improvements were observed in clinical outcomes and reductions in TNF-alpha expression, with increased mucosal *Bifidobacterium* levels. The study concluded that synbiotics effectively improved clinical symptoms in active CD [8]. Despite these promising findings, some studies reported limited or no efficacy. A study by Prantera et al. (2002) assessed the efficacy of *Lactobacillus* GG supplementation and reported modest improvement in maintaining remission in CD [9]. Marteau et al. (2006) conducted a trial evaluating *Lactococcus lactis* expressing human interleukin-10 in patients with mild-to-moderate CD, revealing no significant clinical benefit, although the probiotic was well tolerated [10]. On another note, Sokol et al. (2008) emphasized the strain-specific effects of probiotics, suggesting that a careful selection of bacterial strains is essential for achieving therapeutic success in CD [11]. The variability in clinical outcomes underscores the need for further large-scale, well-designed trials to elucidate the mechanisms of action and identify optimal probiotic strains, dosages, and treatment durations for CD, taking into consideration the heterogeneity of the gene–environment–microbiome conditions between different ethnicities.

In Lebanon, the prevalence of CD has been documented in several studies. Abdul-Baki et al. (2007) reported an age-adjusted prevalence of 53.1 per 100,000 people for CD, with a mean annual incidence of 1.4 per 100,000 people. The mean age at diagnosis was 28.8 years, with a slight female predominance [12]. More recent studies suggest an increasing trend in the incidence and prevalence of CD in Lebanon over the past two decades. It changed by 198.5% between 1990 and 2019 from 65 (54–80) to 194 (161–238), respectively [13]. Lebanese researchers have contributed to understanding CD through various studies. For instance, a cross-sectional study assessed the health-related quality of life (HRQoL) among Lebanese patients with IBD, including CD, highlighting the disease’s negative impact on HRQoL [14]. Another study examined self-reported food intolerances, dietary supplement use, and malnutrition among Lebanese patients with chronic IBD, providing insights into the nutritional challenges faced by this population [15]. To our knowledge, there have been no prior clinical trials examining the effects of probiotic supplementation on the progression of the disease, nutritional intake, and the quality of life of patients with CD in Lebanon. Therefore, this study seeks to evaluate the short-term (2 months) effects of a dietary supplement containing *Lactobacilli*, *Bifidobacteria*, and *Lactococcus bacillus* on disease progression, remission, quality of life, and nutritional intake in Lebanese patients with CD.

## 2. Methodology

### 2.1. Study Design

The study was a multicenter, randomized, single-blinded controlled trial conducted in Al Zahraa University Medical Center’s endoscopy unit and at Badaro Endoscopic Center, Moarbes Hospital, Beirut, Lebanon, from 1 April 2024 to 1 August 2024. The trial identifier on Clinical.Trial.gov is the following: NCT06392061. Recruitment, prescreening, screening, enrollment, and protocol implementation were performed in both centers.

### 2.2. Study Drug

In the concentration of 4.5 milliard CFU/capsule, the probiotic used in the current study contains naturally occurring bacteria in the following proportions: 37.5% *Lactobacillus acidophilus*, 25% *Lactococcus bacillus*, and 37.5%. *Bifidobacterium bifidum*. For a period of 8 weeks, patients in the intervention group were instructed to take one tablet per day of the probiotic supplement [Trilac, provided by Surveal Pharma, Bruxelles, Belgium]. The randomization and allocation sequence were implemented by a research assistant at both centers. Allocation was based on a simple randomization approach, was concealed, and all participants were blinded to drug assignment, until trial and data entry completion.

### 2.3. Study Participants

The contact information, ages, and disease subtypes of 65 patients diagnosed with IBD were gathered and compiled into an Excel spreadsheet using a retrospective approach based on medical files. In total, 59 patients out of 65 were contacted, informed about the study and its two visits, and invited to participate. Of these, 31 agreed to participate in the first interview. Those who declined participation cited reasons such as living abroad, lack of interest in the study, political instability, and some patients’ unwillingness to return or continue the probiotic administration. The final retention was for 21 patients with CD. These study participants were randomly assigned into two groups by the investigators using simple randomization techniques with a series of sealed envelopes. In total, 10 individuals were in the control group (n = 10) and 11 in the intervention group (n = 11).

### 2.4. Study Visits and Measurements

At the initial visit called T0, all patients underwent a clinical examination by the medical team to determine their eligibility. Two research assistants collected demographic information, assessed disease status, and documented allergies, food intolerances, and dietary beliefs regarding CD, as well as lifestyle and dietary patterns during and outside of flare episodes. They also recorded medications and supplements and administered the World Health Organization Quality of Life-BREF (WHOQOL-BREF) [16] instrument which evaluates the quality of life across four key domains: Physical Health, which encompasses energy levels, fatigue, sleep quality, mobility, daily activities, pain, and dependence on medical treatments; Psychological Health, focusing on mental and emotional well-being, including self-esteem, body image, positive and negative feelings, spirituality, and concentration; Social Relationships, which assess personal relationships, social support, and sexual activity; and Environment, examining living conditions, access to resources, safety, transportation, financial stability, and opportunities for leisure activities. Moreover, participants’ intake of energy, macronutrients, and micronutrients was assessed using a 24 h recall tool for three days. The mean of the three days was analyzed using NutritionistPro software (version 7.9; Axxya Systems, based in the United States). These data were collected both at baseline and at the end of the 8-week study period. The Subjective Global Assessment (SGA) questionnaire was also utilized to assess the risk of malnutrition among patients during both study periods. Anthropometric and body composition measurements were conducted at the beginning and end of the study using the Biospace/InBody 770 device (Alpha-Tech, Serial Number: C71400242, Republic of Korea). After two months of weekly follow-ups conducted by the research assistants, patients from both the control and intervention groups were contacted based on their initial visit dates (T0). In the intervention group, participants who had completed the full course of 60 tablets were invited for a second visit called (T1). Due to the small sample size, we were able to closely monitor each participant and follow up with them daily to ensure they took their prescribed dose. During this follow-up, a medical survey was administered to assess disease improvement, clinical response, the number of flares and hospitalizations, changes in medication, and quality of life (using the WHOQOL-Bref). For the intervention group, adherence data and any adverse events over the past two months were also collected. A nutritional survey assessed changes in lifestyle, food intake, and food intolerance patterns following two months of supplementation with probiotics. Additionally, the SGA, anthropometric and body composition measurements, and 3-day 24 h recalls were repeated for each participant.

## 3. Statistical Tests

We employed descriptive statistics, parametric tests (including paired and independent *t*-tests, and the chi-squared test), non-parametric tests, and analysis of variance (ANOVA) to assess differences between and within treatment groups. Regarding the probiotic effect and the differences between the two phases of the study (T0 and T1), the following occurred:

1. A paired *t*-test was used to investigate the difference between T0 and T1 within each study arm.
2. An ANOVA test was added in the revised manuscript to include a comparison between the study arms regarding differences at T0 and T1.

In terms of confounders, baseline disease severity, dietary habits, and other relevant factors were adjusted for using multivariable regression models to ensure that the observed effects were independent of these potential confounders.

## 4. Inclusion/Exclusion Criteria

The main inclusion criteria for the study were being Lebanese outpatients with a histological diagnosis of CD for at least six months prior to the trial, aged between 18 and 65 years, having stable inactive disease, and not using any probiotics within two months prior to or during the study period. The exclusion criteria included pregnant or lactating women, individuals aged under 18 or above 65 years, patients who had used probiotics within the preceding two months or during the study period, and patients with active disease.

## 5. Ethical Approval

The study was approved by the Ethics Committee at Al Zahraa Hospital University Medical Center (ZHUMC-5 August 2023–6 August 2024), and each participant provided written consent after a thorough explanation of the study procedures and probiotic safety.

## 6. Results

### 6.1. Characteristics of the Study Participants

#### 6.1.1. Sociodemographic Characteristics at Baseline

Our results indicate that 60% of participants in the control group and 54% in the intervention group were male. Over 50% of the population was married, with more than 60% of them having children. A higher percentage of unemployed participants was observed in the intervention group compared to the control group (54% vs. 10%; *p* = 0.03). Regarding monthly income, no significant differences were found between the two study groups (*p* = 0.9) (Table 1).

#### 6.1.2. Medical and Anthropometric Characteristics of Study Participants at T0

There were no significant differences regarding the medical and health characteristics of the study population except for the self-reported allergies that were significantly different between both study groups (*p* = 0.04). The control group was more allergic compared to the other one with no specific type of allergy. Most participants in both groups had been diagnosed within the past 1 to 5 years, and their diseases were severe but inactive. Patients self-reported experiencing flares less than three times per year, with a low frequency of hospitalizations (Table 2). As for the anthropometric measurements, at the baseline level, there were no significant differences in the anthropometric and body composition characteristics between both study groups except for the BMI and the arm muscle circumference, which were higher in the intervention group compared to the other one (*p* = 0.01 and *p* = 0.03, respectively) (Table 3).

#### 6.1.3. Dietary Beliefs and Lifestyle Patterns of Participants at T0

The dietary beliefs and lifestyle patterns were similar between the two study groups, particularly regarding the feeding mode, avoidance of specific foods, fear of dining out, regularity in meal planning and eating patterns, and engagement in physical activity. Regarding dieting patterns, over 60% of participants in both study groups reported not following a specific diet, while more than 25% followed a diet based on their own perspectives (Table S1). Regarding dietary supplements, more than 40% of participants in both study groups reported taking supplements regularly, with no significant difference between the groups (*p* = 0.2). The most common sources of dietary supplement prescriptions were medical healthcare professionals (60%) and self-prescription (27%) (Table S2).

#### 6.1.4. Nutritional Intake of Participants at T0

At baseline, based on the 24 h recall, the mean intake of protein, EPA, DHA, sodium, calcium, iron, riboflavin, niacin, zinc, selenium, soluble fiber, insoluble fiber, maltose, and sugar alcohols was significantly higher in the intervention group compared to the control group. Conversely, the intake of vitamin D, chromium, and caffeine was lower in the intervention group compared to the control participants (Table S3).

#### 6.1.5. Assessment of the Quality of Life of Participants at T0

At baseline, no statistical differences in QOL outcomes were observed between both study groups (Table 4).

### 6.2. Findings After Two Months of Probiotic Supplementation at T1

#### 6.2.1. Remission and Diseases Course at T1

The comparison of symptoms, flares, hospitalization, and adverse events occurrence between the control and intervention group after 2 months of supplementation is presented in Table 5. After two months of probiotic supplementation, a similar percentage of participants in both study groups experienced flares (approximately 30%; *p* = 0.8). Most participants were not admitted to any hospital, had normal stool frequency, and reported stool texture alternating between soft and loose (*p* > 0.05). Rectal bleeding was uncommon among participants. However, abdominal pain, primarily at the central abdomen level, was frequently reported in both study groups (*p* = 0.6), with most participants not requiring steroids for pain management (Table 5). Approximately 36% of participants in the intervention group reported either a decrease or an increase in appetite. However, this difference was not significant between the groups. Other changes regarding current treatments did not change also between study groups (Table 5).

As for the adverse effects of the probiotics, half the participants in the intervention group reported no adverse events. However, the other half were facing constipation (9.1%), headache and colic and abdominal pain (9.1%), nausea (18.2%), and streaks of blood associated with bloating (9.1%) (Table 5).

#### 6.2.2. Quality of Life (QOL) Among Participants

Table S4 shows the differences between both study groups regarding QOL where both groups were similar at T1 (*p* > 0.5 for all the QOL items). However, according to Table 6 and Table 7, a notable significant difference between both study periods (T0 and T1) was seen among participants in the intervention group, especially in physical, psychological, and environmental health, which was not the case among the control group’s participants, who had amelioration in their psychological health only (Table 6 and Table 7).

#### 6.2.3. Participants’ Dietary Patterns, Malnutrition, Lifestyle, and Anthropometric Differences at T1

No significant differences were seen among each study arm at T1 regarding dietary patterns, malnutrition, lifestyle (Table S5), anthropometric measurements, and body composition (Tables S6 and S7). Regarding anthropometric measurements, no modifications were seen in the control group after two months of supplementation (Table S8). However, participants in the intervention group witnessed an increase in their body weight (*p* = 0.01), BMI (*p* = 0.01), body fat mass (*p* = 0.04), and arm muscle circumference (*p* = 0.01) (Table 8). When comparing the changes in anthropometric measurements between groups at T1, we observed a significant increase in body weight (control: 66 ± 13 vs. 78 ± 12; *p* = 0.04), BMI (24 ± 3 vs. 29 ± 3; *p* = 0.006), BMF (18 ± 9 vs. 26 ± 9; *p* = 0.049), and AMC (26 ± 3 vs. 29 ± 3; *p* = 0.017).

As for the nutrient’s intake, when comparing both study groups at T1, the nutrients were similar except for calcium, riboflavin, and folate, which were higher in the intervention group compared to the control (*p* = 0.01, *p* = 0.04, *p* = 0.013, respectively) (Table S9). However, after two months of supplementation, among the intervention group, between T0 and T1, folate increased (*p* = 0.02), dietary fiber increased (*p* = 0.01), total sugar increased (*p* = 0.02), and caffeine doubled (*p* = 0.01). As for the control, energy, EPA, Ca, riboflavin, niacin, Magnesium, and total sugar increased significantly between T0 and T1 (*p* = 0.04, *p* = 0.04, *p* = 0.001, *p* = 0.02, *p* = 0.03, *p* = 0.02, and *p* = 0.008, respectively) (Table 9). When comparing both study groups at T1, we observed an increase in calcium intake among the intervention group only (*p* = 0.04). Multivariate linear regression analyses, adjusting for age, BMI, and the baseline values of all study variables, showed no difference at 2 months between the two study arms.

## 7. Discussion

Our study found promising effects of probiotic supplementation in patients with Crohn’s Disease (CD), particularly in quality of life (QOL) improvements across physical, psychological, and environmental domains. The intervention group showed significant enhancements in all three domains, while the control group demonstrated improvements only in psychological health. This suggests that probiotics may offer a more comprehensive benefit to overall well-being in CD patients. The improvements observed in physical and environmental QOL are particularly important, as they reflect broader aspects of daily functioning and life satisfaction that are often severely impacted in chronic conditions like CD. These findings are consistent with research emphasizing the complex interplay between the gut microbiome and health outcomes in CD. The physical QOL improvements observed in our study may indicate that probiotics help mitigate some of the physical discomforts associated with CD, such as fatigue, abdominal pain, or malnutrition. In fact, studies have demonstrated that probiotics, especially certain strains like *Lactobacillus* and *Bifidobacterium*, can play a role in reducing gut inflammation, improving nutrient absorption, and even regulating intestinal motility [17,18]. For CD patients, improving physical functioning is crucial, as it directly affects their ability to carry out daily tasks, engage in social activities, and maintain independence. This aligns with the findings of a recent systematic review that analyzed the safety and efficacy of various probiotics in treating CD, revealing that while some probiotics like kefir and *Lactobacillus thermophilus* show promise in reducing inflammation and improving quality of life, the overall evidence for their efficacy in achieving remission is limited. This systematic review that encompasses 16 studies noted that probiotics, particularly kefir, reduced inflammation and significantly improved QOL in CD patients by alleviating symptoms like diarrhea and abdominal discomfort, independently of remission [19]. Psychological well-being is another domain where probiotics have shown potential benefits. Our study’s findings of significant psychological improvements in the intervention group further support the growing body of evidence suggesting that the gut–brain axis plays a critical role in mental health. Emerging research has highlighted that patients with CD are at increased risk of mental health disorders like depression and anxiety, likely due to the chronic nature of the disease, the stress of managing flare-ups, and the impact of gastrointestinal symptoms on daily life [20,21]. A study by Clarke et al. (2020) demonstrated that gut microbiota imbalances in patients with IBD, including CD, are linked to mental health disorders, and probiotics may help restore microbial balance, which in turn improves mood and reduces anxiety [22]. This finding aligns with studies showing that probiotics, such as *Lactobacillus* and *Bifidobacterium*, have mood-enhancing effects by modulating gut-derived signals that influence brain function [23]. Thus, improving psychological well-being can have a significant impact on CD patients, helping them cope with the emotional and mental toll of living with a chronic illness. The environmental domain of QOL in our study, which reflects the patient’s perception of their social, work, and home environment, also improved significantly in the intervention group. This is an important aspect of chronic disease management, as CD often leads to social isolation due to symptom flare-ups, hospitalizations, or dietary restrictions. Probiotics’ potential role in reducing gastrointestinal symptoms and promoting better gut health may indirectly improve patients’ ability to participate in social and work activities, further enhancing their overall life satisfaction and integration into their environment. This finding is supported by research indicating that improving gut health and reducing CD symptoms can foster greater social engagement and enhance quality of life in patients with CD [22,23]. Moreover, our study found significant increases in body weight, BMI, body fat mass, and arm muscle circumference in the intervention group. These results suggest that probiotics may support better nutritional status in CD patients, who often struggle with malnutrition due to impaired nutrient absorption and increased metabolic demands during disease flare-ups. Several studies have highlighted the potential of probiotics in supporting nutrient absorption, improving gut permeability, and enhancing intestinal barrier function, all of which are crucial for weight maintenance and overall health in CD patients [18,24]. For example, *Bifidobacterium* lactis has been shown to help mitigate excessive weight loss and inflammation in animal models of CD, while *Lactobacillus* strains can help increase the production of short-chain fatty acids, which are beneficial for gut health and energy metabolism [25,26]. These changes in body composition may also improve patients’ energy levels and contribute to better physical functioning, supporting the improvements in physical QOL observed in our study.

Despite these positive findings, our study also revealed that probiotic supplementation did not significantly affect disease-specific outcomes such as symptoms, flare-ups, hospitalization rates, or adverse events between the intervention and control groups after two months. This lack of impact on disease activity is consistent with several systematic reviews and meta-analyses, which have questioned the efficacy of probiotics in managing CD symptoms (flares, hospitalization, etc.), especially when multi-strain formulations like *Bifidobacterium* sp. are used [27,28]. Although some studies have highlighted specific probiotics, such as *Lactobacillus thermophilus* and *Bifidobacterium longum*, as potentially beneficial for managing symptoms, the evidence remains inconclusive and context dependent [19]. It is possible that the probiotic strains used in our study did not exert the necessary effects on gut inflammation or disease activity, underscoring the need for further research to identify which strains may have therapeutic potential for symptom management. Additionally, the short duration of the clinical trial may have limited the opportunity for these probiotic strains to fully ameliorate gut inflammation or disease activity, suggesting that longer-term studies are needed to better assess their therapeutic potential for symptom management. This can explain the negative findings shown when applying multivariate linear regression analyses. Additionally, while adverse effects were generally mild, they were reported by half of the participants in the intervention group and included constipation, headache, nausea, and bloating. While these effects are typically transient and manageable, they highlight the variability in individual responses to probiotics, which may depend on factors like gut microbiota composition and the specific probiotic strain used [29]. It is important to note that probiotics are not intended to replace conventional treatments for CD, but rather to be considered as a complementary approach

## 8. Strength and Limitations

The study’s strengths include its multicenter design, which enhances generalizability, and its randomized, single-blind approach, which minimizes bias and strengthens internal validity. Additionally, the comprehensive use of various outcome measures, such as the WHOQOL-BREF for quality of life, the SGA for malnutrition risk, and anthropometric data, provides a well-rounded assessment of the effects of probiotics on CD patients. However, the study has limitations, including a small sample size of only 21 patients and a short duration of 8 weeks, limiting the ability to observe long-term effects. Despite the small sample size, we have ensured that the statistical power of the study remains sufficient for the analysis. Recruitment and retention issues also introduced potential bias, with many potential participants declining to join, and the exclusion of active disease patients means the findings may not be applicable to those with more active conditions.

## 9. Conclusions

In conclusion, while our study demonstrated that probiotics may offer significant improvements in quality of life, mental health, and body composition in CD patients, they did not have a significant impact on disease-specific outcomes such as symptom management or hospitalization rates. These results suggest that probiotics may have a more indirect role in managing CD, possibly through improving overall well-being, psychological health, and nutritional status. Given the complexity of CD and its multifactorial nature, probiotics may be most beneficial when used as part of a broader, individualized treatment strategy that includes medication, dietary adjustments, and other lifestyle interventions. Further large-scale, well-designed, randomized controlled trials are needed to better understand the precise mechanisms by which probiotics can influence CD and to identify which strains or formulations are most effective in improving disease outcomes.

## Figures and Tables

**Table 1:** Sociodemographic characteristics of the study participants at T0.

		Group				*p*-Value
		Control		Intervention		
		N	%	N	%	
Gender	Female	4	40.0%	5	45.5%	0.8
	Male	6	60.0%	6	54.5%	
Marital status	Divorced	1	10.0%	1	9.1%	0.8
	Married	5	50.0%	7	63.6%	
	Single	4	40.0%	3	27.3%	
Have children	No	4	40.0%	4	36.4%	0.8
	Yes	6	60.0%	7	63.6%	
Profession	Employed	9	90.0%	5	45.5%	0.03
	Unemployed	1	10.0%	6	54.5%	
Monthly income	>20 million LBP	7	70.0%	7	63.6%	0.9
	10–15 million LBP	1	10.0%	2	18.2%	
	15–20 million LBP	1	10.0%	1	9.1%	
	5–10 million LBP	1	10.0%	1	9.1%	

**Table 2:** Medical and health characteristics of the study population at T0.

		Study Group				*p*-Value
		Control		Intervention		
		N	%	N	%	
Alcohol	No	8	80.0%	9	81.8%	0.9
	Yes	2	20.0%	2	18.2%	
Health conditions	1 disease	3	30.0%	4	36.4%	0.7
	2 diseases	1	10.0%	0	0.0%	
	>2 diseases	1	10.0%	1	9.1%	
	None	5	50.0%	6	54.5%	
Medication intake	1 medication	3	30.0%	2	18.2%	0.8
	>2 medications	1	10.0%	1	9.1%	
	None	6	60.0%	8	72.7%	
Allergies	No	4	40.0%	9	81.8%	0.04
	Yes	6	60.0%	2	18.2%	
Type of allergies	Environmental allergies *	3	30.0%	0	0.0%	0.07
	Environmental allergies coupled with medication allergies	0	0.0%	1	9.1%	
	Food allergies	1	10.0%	0	0.0%	
	Food allergies, environmental allergies, and medication allergies	0	0.0%	1	9.1%	
	Medication allergies	2	20.0%	0	0.0%	
	Not applicable	4	40.0%	9	81.8%	
Time since being diagnosed with CD	1–5 years	5	50.0%	7	63.6%	0.5
	>10 years	5	50.0%	4	36.4%	
Surgery	No	8	80.0%	10	90.9%	0.4
	Yes	2	20.0%	1	9.1%	
CD-related surgery	1 surgery	1	10.0%	1	9.1%	0.5
	2 surgeries	1	10.0%	0	0.0%	
	None	8	80.0%	10	90.9%	
Area of inflammation by CD	Colonic	0	0.0%	1	9.1%	0.5
	Ileal	6	60.0%	5	45.5%	
	Ileal, Rectum	0	0.0%	1	9.1%	
	Ileocolonic	4	40.0%	2	18.2%	
	Ileocolonic, Rectum	0	0.0%	1	9.1%	
	Pan colon	0	0.0%	1	9.1%	
Disease severity	Moderate	3	30.0%	2	18.2%	0.5
	Severe	7	70.0%	9	81.8%	
Montréal classification	Unknown	0	0.0%	1	9.1%	0.4
	A1 L3 B1	1	10.0%	0	0.0%	
	A2 L1 B1	2	20.0%	2	18.2%	
	A2 L1 B2	0	0.0%	1	9.1%	
	A2 L2 B3p	0	0.0%	1	9.1%	
	A2 L3 B1	3	30.0%	2	18.2%	
	A2 L3 B2	0	0.0%	1	9.1%	
	A3 L1 B1	2	20.0%	1	9.1%	
	A3 L1 B1p	0	0.0%	1	9.1%	
	A3 L1 B2	2	20.0%	0	0.0%	
	A3 L3 B3p	0	0.0%	1	9.1%	
Flares per year	≤3	9	90%	9	81.9%	0.7
	>4	1	10%	2	18.2%	
Hospitalization per year	0	9	90.0%	8	72.7%	0.2
	1	0	0.0%	1	9.1%	
	2	0	0.0%	2	18.2%	
	3	1	10.0%	0	0.0%	
Current treatment	5-ASA	1	10.0%	0	0.0%	0.9
	5-ASA, Biological (Anti-TNF)	1	10.0%	2	18.2%	
	Biological (Anti-TNF)	3	30.0%	4	36.4%	
	Corticosteroid, Biological (Anti-TNF)	1	10.0%	1	9.1%	
	Immunosuppressant, Biological (Anti-TNF)	2	20.0%	2	18.2%	
	None	2	20.0%	2	18.2%	

* pollen, dust, etc.

**Table 3:** Anthropometric and body composition characteristics of both study groups at T0.

	Study Group				
	Control		Intervention		
	Mean	Standard Deviation	Mean	Standard Deviation	*p*-Value
Weight before the disease (kg)	68	17	68	14	0.9
Current weight	65	14	77	13	0.05
Height	167	10	167	10	0.9
Body Mass Index (BMI)	23	4	28	3	0.01
Waist Circumference (cm)	86	11	92	31	0.5
TBW (Total body water) (L)	35	8	38	9	0.4
ICW (Intracellular Water) (L)	22	5	24	6	0.4
ECW (Extracellular Water) (L)	13	3	15	3	0.4
Protein (kg)	9	2	10	3	0.4
Minerals	3	1	4	1	0.3
BFM (Body Fat Mass) (kg)	18	9	25	8	0.07
SLM (Soft Lean Mass) (kg)	45	11	49	12	0.4
FFM (Fat Free Mass) (kg)	48	11	52	12	0.4
SMM (Skeletal Muscle Mass) (kg)	26	7	29	8	0.4
PBF (Percent Body Fat) (%)	27	12	32	11	0.3
Basal Metabolic Rate	1400	248	1496	269	0.4
WHR (Waist–Hip Ratio)	1	0	1	0	0.06
AMC (Arm Muscle Circumference)	26.04	2.8	28.8	2.7	0.03

**Table 4:** Comparison of QOL between control and intervention groups at T0.

	Group	N	Mean	Std. Deviation	*p*-Value
Raw Score for Physical Health	Control	10	22.50	3.274	0.4
	Intervention	11	22.91	4.253	
Transformed Score (0–100) for Physical Health	Control	10	55.80	11.905	0.4
	Intervention	11	57.09	15.827	
Raw Score for Psychological	Control	10	21.00	1.764	0.09
	Intervention	11	20.55	3.078	
Transformed Score (0–100) for Psychological	Control	10	62.70	7.319	0.1
	Intervention	11	60.91	13.531	
Raw Score for Social Relationships	Control	10	10.90	1.370	0.7
	Intervention	11	11.36	1.748	
Transformed Score (0–100) for Social Relationships	Control	10	64.90	13.093	0.7
	Intervention	11	69.27	14.670	
Raw Score for Environment	Control	10	24.70	4.448	0.8
	Intervention	11	24.09	4.784	
Transformed Score (0–100) for Environment	Control	10	51.40	14.362	0.5
	Intervention	11	60.36	11.595	

**Table 5:** Comparison of symptoms, flares, hospitalization, and adverse events occurrence between control and intervention group after 2 months of supplementation.

		Study Group				
		Control		Intervention		
		N	N %	N	N %	*p*-Value
During the last 2 months, did you experience any flare?	No	7	70.0%	8	72.7%	0.8
	Yes	3	30.0%	3	27.3%	
Number of flares during the last 2 months	≤3	9	90.0%	9	72.7%	0.5
	>4	1	10.0%	2	9.1%	
Number of hospitalizations due to disease during the last 2 months	0	9	90.0%	11	100.0%	0.2
	1	1	10.0%	0	0.0%	
Perianal disease	No	10	100.0%	8	72.7%	0.07
	Patients already have one prior to the study	0	0.0%	3	27.3%	
Current stool frequency	1–2 stools more than normal	0	0.0%	2	18.2%	0.3
	3–4 stools more than normal	1	10.0%	1	9.1%	
	Normal number of stools	9	90.0%	8	72.7%	
The texture of bowel motions mostly seen	Alternating between soft and loose stool	6	60.0%	7	63.6%	0.8
	Formed stool	1	10.0%	2	18.2%	
	Loose stool	1	10.0%	1	9.1%	
	Watery stool	2	20.0%	1	9.1%	
Current rectal bleeding	No blood seen	10	100.0%	10	90.9%	0.3
	Streaks of blood with stool less than one-half of the time	0	0.0%	1	9.1%	
Physician’s global assessment	Mild disease	0	0.0%	1	9.1%	0.4
	Moderate disease	5	50.0%	6	54.5%	
	Normal	0	0.0%	1	9.1%	
	Severe disease	5	50.0%	3	27.3%	
Abdominal pain	No	5	50.0%	5	45.5%	0.8
	Yes	5	50.0%	6	54.5%	
Description of the location of abdominal pain	Central abdomen	3	30.0%	4	36.4%	0.6
	Lower abdomen	1	10.0%	0	0.0%	
	Not applicable	5	50.0%	5	45.5%	
	Upper abdomen	1	10.0%	2	18.2%	
Steroids for pain	No	6	60.0%	8	72.7%	0.5
	Not applicable, no symptoms have occurred	3	30.0%	3	27.3%	
	Yes	1	10.0%	0	0.0%	
Change in appetite	No change	6	60.0%	4	36.4%	0.5
	Decrease in appetite	2	20.0%	4	36.4%	
	Increase in appetite	2	20.0%	3	27.3%	
Perianal fissure	Decrease in pus leakage	0	0.0%	1	9.1%	0.3
	Not applicable, patients do not have a perianal disease modifier	10	100.0%	9	81.8%	
	Patients never experienced pus leakage even prior to study	0	0.0%	1	9.1%	
Surgery	No	10	100.0%	10	90.9%	0.3
	Yes	0	0.0%	1	9.1%	
Current treatments	5-ASA	1	10.0%	0	0.0%	0.7
	5-ASA, Biological (Anti-TNF)	1	10.0%	2	18.2%	
	Biological (Anti-TNF)	3	30.0%	4	36.4%	
	Corticosteroid, Biological (Anti-TNF)	1	10.0%	0	0.0%	
	Immunosuppressant, Biological (Anti-TNF)	2	20.0%	3	27.3%	
	None	2	20.0%	2	18.2%	
Adverse events	No	0	0.0%	3	54.5%	<0.001
	Yes	0	0.0%	5	45.5%	
Self-reported adverse events	Constipation	0	0.0%	1	9.1%	0.01
	Headache, colic, abdominal pain	0	0.0%	1	9.1%	
	Nausea	0	0.0%	2	18.2%	
	No adverse events occurred	0	0.0%	6	54.5%	
	Streaks of blood seen, bloating	0	0.0%	1	9.1%	
Adverse events resolved	No adverse events occurred	0	0.0%	6	54.5%	<0.001
	Yes	0	0.0%	5	45.5%	

**Table 6:** QOL modifications within control group between T0 and T1.

	Mean	N	Std. Deviation	*p*-Value
Raw Score for Domain 1 (Physical Health) at T0	22.50	10	3.274	0.3
Raw Score for Domain 1 (Physical Health) at T1	24.10	10	3.84274	
Transformed Score (0–100) for Domain 1 (Physical Health) at T0	55.80	10	11.905	0.3
Transformed Score (0–100) for Domain 1 (Physical Health) at T1	61.40	10	12.929	
Raw Score for Domain 2 (Psychological) at T0	21.00	10	1.764	0.03
Raw Score for Domain 2 (Psychological) at T1	22.90	10	3.281	
Transformed Score (0–100) for Domain 2 (Psychological) at T0	62.70	10	7.319	0.04
Transformed Score (0–100) for Domain 2 (Psychological) at T1	70.50	10	12.774	
Raw Score for Domain 3 (Social Relationships) at T0	10.90	10	1.370	0.6
Raw Score for Domain 3 (Social Relationships) at T1	11.20	10	2.044	
Transformed Score (0–100) for Domain 3 (Social Relationships) at T0	64.90	10	13.093	0.5
Transformed Score (0–100) for Domain 3 (Social Relationships) at T1	68.80	10	17.492	
Raw Score for Domain 4 (Environment) at T0	24.70	10	4.448	0.7
Raw Score for Domain 4 (Environment) at T1	25.00	10	3.018	
Transformed Score (0–100) for Domain 4 (Environment) at T0	51.40	10	14.362	0.6
Transformed Score (0–100) for Domain 4 (Environment) at T1	54.40	10	9.732	

**Table 7:** QOL modifications within intervention group between T0 and T1.

	Mean	N	Std. Deviation	*p*-Value
Raw Score for Domain 1 (Physical Health) at T0	22.91	11	4.253	0.03
Raw Score for Domain 1 (Physical Health) at T1	25.8182	11	3.06001	
Transformed Score (0–100) for Domain 1 (Physical Health) at T0	57.09	11	15.827	0.03
Transformed Score (0–100) for Domain 1 (Physical Health) at T1	67.82	11	11.116	
Raw Score for Domain 2 (Psychological) at T0	20.55	11	3.078	0.04
Raw Score for Domain 2 (Psychological) at T1	22.00	11	2.720	
Transformed Score (0–100) for Domain 2 (Psychological) at T0	60.91	11	13.531	0.07
Transformed Score (0–100) for Domain 2 (Psychological) at T1	66.55	11	10.885	
Raw Score for Domain 3 (Social Relationships) at T0	11.36	11	1.748	0.7
Raw Score for Domain 3 (Social Relationships) at T1	11.55	11	2.067	
Transformed Score (0–100) for Domain 3 (Social Relationships) at T0	69.27	11	14.670	0.8
Transformed Score (0–100) for Domain 3 (Social Relationships) at T1	70.36	11	17.761	
Raw Score for Domain 4 (Environment) at T0	24.09	11	4.784	0.003
Raw Score for Domain 4 (Environment) at T1	27.36	11	3.529	
Transformed Score (0–100) for Domain 4 (Environment) at T0	60.36	11	11.595	0.7
Transformed Score (0–100) for Domain 4 (Environment) at T1	62.18	11	11.161	

**Table 8:** Comparison within groups between T0 and T1 regarding changes in anthropometric measurements.

			Mean	N	Std. Deviation	*p*-Value
Control	Waist Circumference (cm)	WC at T1	90.30	10	17.651	0.5
		WC at T0	85.65	10	10.975	
	Current weight (kg)	Current weight at T1	65.91	10	13.498	0.3
		Current weight at T0	65.35	10	13.877	
	Body Mass Index (kg/m^2^)	BMI at T1	23.52	10	3.384	0.3
		BMI at T0	23.31	10	3.604	
	Total body water (L)	TBW at T1	35.28	10	8.216	0.4
		TBW at T0	35.02	10	8.454	
	Intracellular Water	ICW at T1	21.78	10	5.212	0.4
		ICW at T0	21.62	10	5.357	
	Extracellular Water	ECW at T1	13.50	10	3.013	0.4
		ECW at (L)_T0	13.40	10	3.104	
	Protein (kg)	P at T1	9.40	10	2.244	0.4
		P at T0	9.33	10	2.298	
	Minerals (kg)	M at T1	3.41	10	0.716	0.1
		M at T0	3.33	10	0.780	
	Body Fat Mass (BMF)	BFM at T1	17.83	10	8.570	0.6
		BFM at T0	17.67	10	9.200	
	Soft Lean Mass at T0	SLM at T1	45.28	10	10.605	0.4
		SLM at T0	44.92	10	10.905	
	Fat Free Mass (kg)	FFM at T1	48.08	10	11.156	0.4
		FFM at T0	47.68	10	11.496	
	Skeletal Muscle Mass (kg)	SMM at T1	26.41	10	6.835	0.4
		SMM at T0	26.18	10	6.993	
	Percent Body Fat (%)	PBF at T1	26.83	10	11.117	0.8
		PBF (%) at T0	26.67	10	12.081	
	Basal Metabolic Rate	BMR at T1	1407.90	10	241.224	0.4
		Basal Metabolic Rate at T0	1400.20	10	248.406	
	Waist–Hip Ratio	WHR at T1	0.89	10	0.073	0.2
		WHR at T0	0.88	10	0.079	
	Arm Muscle Circumference	AMC at T1	26.20	10	2.930	0.2
		AMC at T0	26.04	10	2.866	
Intervention	Waist Circumference (cm)	WC at T1	84.27	11	15.533	0.5
		WC at T0	92.05	11	30.852	
	Current weight (kg)	Current weight at T1	78.06	11	11.998	0.01
		Current weight at T0	77.18	11	12.791	
	Body Mass Index (kg/m^2^)	BMI at T1	28.08	11	3.331	0.01
		BMI at T0	27.75	11	3.457	
	Total body water (L)	TBW at T1	38.48	11	8.643	0.4
		TBW at T0	38.25	11	9.078	
	Intracellular Water	ICW at T1	23.85	11	5.575	0.3
		ICW at T0	23.67	11	5.906	
	Extracellular Water	ECW at T1	14.63	11	3.084	0.6
		ECW at (L)_T0	14.57	11	3.201	
	Protein (kg)	P at T1	10.33	11	2.405	0.2
		P at T0	10.23	11	2.551	
	Minerals (kg)	M at T1	3.65	11	0.799	0.7
		M at T0	3.64	11	0.814	
	Body Fat Mass (BMF)	BFM at T1	25.61	11	8.508	0.04
		BFM at T0	25.07	11	8.446	
	Soft Lean Mass at T0	SLM at T1	49.44	11	11.187	0.3
		SLM at T0	49.08	11	11.754	
	Fat Free Mass (kg)	FFM at T1	52.45	11	11.825	0.3
		FFM at T0	52.11	11	12.425	
	Skeletal Muscle Mass (kg)	SMM at T1	29.13	11	7.270	0.2
		SMM at T0	28.85	11	7.676	
	Percent Body Fat (%)	PBF at T1	32.30	10	11.287	0.3
		PBF (%) at T0	31.95	10	11.435	
	Basal Metabolic Rate	BMR at T1	1502.91	11	255.381	0.4
		Basal Metabolic Rate at T0	1496.09	11	268.613	
	Waist–Hip Ratio	WHR at T1	0.97	11	0.083	0.4
		WHR at T0	0.95	11	0.093	
	Arm Muscle Circumference	AMC at T1	29.36	11	2.609	0.01
		AMC at T0	28.87	11	2.789	

**Table 9:** Nutrient modifications between T0 and T1, within groups.

		Mean	N	Std. Deviation	*p*-Value
Control	E at T1	3583.20	10	2711.510	0.04
	Energy at T0	1914.56	10	1006.947	
	Protein at T1	106.24	10	83.701	0.1
	Protein at T0	61.17	10	21.173	
	CHO at T1	428.81	10	334.805	0.08
	CHO at T0	224.65	10	135.339	
	Fat_T1	165.29	10	143.726	0.05
	Fat_T0	85.68	10	52.653	
	CT at T1	260.87	10	272.914	0.6
	CT at T0	203.51	10	217.583	
	SFA at T1	33.80	10	27.722	0.06
	SFA at T0	16.53	10	7.705	
	MUFA at T1	67.55	10	67.535	0.06
	MUFA at T0	30.97	10	25.698	
	PUFA at T1	33.44	10	40.844	0.2
	PUFA at T0	19.03	10	17.137	
	Oleic at T1	63.86	10	64.716	0.06
	Oleic at T0	29.08	10	24.792	
	Linoleic at T1	30.63	10	37.577	0.2
	Linoleic at T0	17.57	10	16.114	
	Linolenic at T1	2.35	10	2.884	0.2
	Linolenic at T0	1.19	10	1.126	
	EPA_T1	0.03	10	0.047	0.04
	EPA_T0	0.00	10	0.000	
	DHA_T1	0.07	10	0.107	0.2
	DHA_T0	0.02	10	0.042	
	TFA_T1	2.37	9	4.412	0.1
	TFA_T0	0.41	9	0.542	
	Na_T1	3205.93	10	2564.807	0.3
	Na_T0	2479.51	10	1094.783	
	K_T1	5265.98	10	4575.630	0.04
	K_T0	2422.27	10	1720.743	
	Vitamin C_T1	74.89	10	58.400	0.1
	Vitamin C_T0	40.29	10	36.187	
	Calcium_T1	567.13	10	200.172	0.001
	Ca_T0	272.85	10	96.770	
	Iron_T1	17.60	10	12.491	0.06
	Iron_T0	10.37	10	4.348	
	Vitamin D_T1	1.41	10	1.379	0.1
	Vitamin D_T0	28.11	10	52.846	
	Thiamin_T1	1.75	10	1.368	0.1
	Thiamin_T0	1.05	10	0.602	
	Riboflavin_T1	1.45	10	0.603	0.02
	Riboflavin_T0	0.92	10	0.434	
	Niacine_T1	29.57	10	21.950	0.03
	Niacin_T0	16.19	10	8.815	
	Folate_T1	459.90	10	313.347	0.06
	Folate_T0	255.46	10	123.158	
	Cobalamin_T1	5.16	10	9.575	0.3
	VitaminB12_T0	2.05	10	1.775	
	Vitamin K_T1	106.74	10	97.499	0.05
	Vitamin K_T0	50.57	10	37.797	
	Mg_T1	380.27	10	274.785	0.02
	Mg_T0	174.33	10	110.775	
	Dietary_fiber_T1	35.51	10	29.493	0.05
	DF_T0	15.21	10	11.502	
	soluble_fiber_T1	0.52	10	1.012	0.1
	SF_T0	0.01	10	0.032	
	soluble_fiber_T1	0.52	10	1.012	0.1
	Insoluble fiber_T0	0.05	10	0.108	
	Crude fiber_T1	2.51	10	1.774	0.05
	Crude fiber _T0	1.30	10	1.206	
	Total sugar_T1	96.85	10	50.206	0.008
	Sugar	35.36	10	34.463	
	Caffeine_T1	109.30	10	78.745	0.1
	Caffeine	68.09	10	114.335	
Intervention	E_T1	3546.39	10	2176.270	0.3
	Energy_T0	2643.91	10	1920.355	
	Protein food_T1	147.75	10	138.190	0.3
	Protein_T0	98.13	10	66.738	
	CHO_T1	391.60	10	165.747	0.2
	CHO_T0	294.83	10	170.266	
	Fat_T1	157.25	10	113.655	0.4
	Fat_T0	121.28	10	110.351	
	CT_T1	493.27	10	487.342	0.5
	CT_T0	347.20	10	357.805	
	SFA_T1	35.11	10	23.317	0.5
	SFA_T0	26.92	10	26.722	
	MUFA_T1	57.55	10	48.353	0.5
	MUFA_T0	43.97	10	49.813	
	PUFA_T1	31.25	10	28.285	0.5
	PUFA_T0	24.70	10	25.621	
	Oleic_T1	53.92	10	45.798	0.5
	Oleic_T0	41.73	10	48.395	
	Linoleic_T1	28.16	10	26.090	0.5
	Linoleic_T0	22.40	10	23.539	
	Linolenic_T1	1.94	10	1.684	0.7
	Linolenic_T0	1.68	10	1.898	
	EPA_T1	0.03	10	0.043	0.5
	EPA_T0	0.02	10	0.042	
	DHA_T1	0.10	10	0.142	0.4
	DHA_T0	0.07	10	0.125	
	TFA_T1	1.06	10	1.128	0.4
	TFA_T0	0.75	10	0.513	
	Na_T1	3931.90	10	2199.225	0.6
	Na_T0	4599.67	10	3903.045	
	K_T1	4524.17	10	3241.576	0.2
	K_T0	3246.41	10	2752.775	
	Vitamin C_T1	70.59	10	32.226	0.3
	Vitamin C_T0	55.70	10	39.745	
	Calcium_T1	1057.45	10	673.200	0.1
	Ca_T0	528.74	10	483.544	
	Iron_T1	19.18	10	10.985	0.2
	Iron_T0	13.78	10	9.648	
	Vitamin D_T1	2.20	10	3.316	0.5
	Vitamin D_T0	1.38	10	2.516	
	Thiamin_T1	1.74	10	0.964	0.3
	Thiamin_T0	1.33	10	0.983	
	Riboflavin_T1	2.00	10	1.291	0.1
	Riboflavin_T0	1.17	10	0.787	
	Niacine_T1	40.69	10	54.931	0.2
	Niacin_T0	22.61	10	19.395	
	Folate_T1	545.36	10	184.765	0.02
	Folate_T0	324.09	10	191.366	
	Cobalamin_T1	8.90	10	17.199	0.2
	Vitamin B12_T0	2.31	10	2.371	
	Vitamin K_T1	181.31	10	270.710	0.4
	Vitamin K_T0	112.74	10	136.389	
	Mg_T1	422.99	10	283.557	0.08
	Mg_T0	243.35	10	195.512	
	Dietary_fiber_T1	34.86	11	21.360	0.01
	DF_T0	22.72	11	14.622	
	soluble_fiber_T1	0.14	11	0.142	0.07
	SF_T0	0.05	11	0.104	
	soluble_fiber_T1	0.14	11	0.142	0.3
	Insoluble fiber_T0	0.27	11	0.469	
	Crude_fiber_T1	1.85	11	1.363	0.2
	Crude fiber_T0	1.52	11	1.320	
	Total_sugar_T1	96.86	11	71.418	0.02
	Sugar_T0	40.33	11	29.183	
	Caffeine_T1	79.23	11	65.732	0.01
	Caffeine_T0	34.52	11	39.255	

## Data Availability

The original contributions presented in the study are included in the article. Further inquiries can be directed to the corresponding author.

## Supplementary Materials

The following supporting information can be downloaded at: https://www.mdpi.com/article/10.3390/nu17040708/s1, Table S1. Dietary beliefs and lifestyle patterns of patients with CD per study group at baseline level; Table S2. Dietary Supplement use by patients with CD per study group at baseline level; Table S3. Comparison between nutrient profiles of both study groups at baseline; Table S4. QALYS between the two study groups after 2 months’ supplementation by probiotics; Table S5. Nutrition status and dietary patterns of the two study groups after two months’ supplementation; Table S6. Anthropometric variation between the two groups after two months’ supplementation; Table S7. Anthropometric variation between groups after two months’ supplementation. Table S8. Dietary patterns, malnutrition, and lifestyle assessment among both groups, each one alone after two months of supplementation; Table S9. Comparisons between study groups regarding nutrient intake after two months of the study.
