# Supplementary material for: Evaluating the Efficacy of Probiotics on Disease Progression, Quality of Life, and Nutritional Status Among Patients with Crohn’s Disease: A Multicenter, Randomized, Single-Blinded Controlled Trial
Source: Nutrients. 2025 Feb 17;17(4):708. doi: 10.3390/nu17040708 (PMC11858769; doi:10.3390/nu17040708)
Supplement: Supplementary file 1 [file nutrients-17-00708-s001.zip › nutrients-3472057-supplementary.pdf]

Table S1. Dietary beliefs and lifestyle patterns of patients with CD per study groups at baseline level.

		Study Group				
		Control		Intervention		p-value
		N	%	N	%	
Is your feeding mode convenient for you?	No	4	40.0%	3	27.3%	0.5
	Yes	6	60.0%	8	72.7%	
To what degree does it influence your quality of life? (on a scale of 1 to 10, 1 = not at all, 10 = completely)	1	0	0.0%	1	9.1%	0.6
	3	2	20.0%	0	0.0%	
	4	2	20.0%	2	18.2%	
	5	4	40.0%	6	54.5%	
	7	1	10.0%	1	9.1%	
	8	1	10.0%	1	9.1%	
Do you believe that certain foods increase the risk of developing CD?	No	1	10.0%	2	18.2%	0.5
	Yes	9	90.0%	9	81.8%	
Do you consider that diet is a trigger of CD flares?	No	1	10.0%	1	9.1%	0.9
	Yes	9	90.0%	10	90.9%	
Do you avoid some food groups to prevent a disease relapse?	No	1	10.0%	3	27.3%	0.3
	Yes	9	90.0%	8	72.7%	
Do you avoid some food groups for fear of worsening the disease flare?	No	2	20.0%	2	18.2%	0.9
	Yes	8	80.0%	9	81.8%	
Do you believe that it would be useful to receive nutritional advice provided by qualified personnel?	No	4	40.0%	1	9.1%	0.9
	Yes	6	60.0%	10	90.9%	
Is your appetite affected during disease remission?	No	2	20.0%	2	18.2%	0.3
	Yes	8	80.0%	9	81.8%	
Is your appetite affected during disease flare?	No	1	10.0%	2	18.2%	0.9
	Yes	9	90.0%	9	81.8%	
Have you modified your dietary habits since CD diagnosis?	No	3	30.0%	2	18.2%	0.5
	Yes	7	70.0%	9	81.8%	
Do you refuse to dine out because of fear that food could worsen your symptoms?	No	3	30.0%	8	72.7%	0.05
	Yes	7	70.0%	3	27.3%	
Are your daily meals regular?	No	4	40.0%	7	63.6%	0.2
	Yes	6	60.0%	4	36.4%	
Number of meals per day	1	3	30.0%	1	9.1%	0.1
	2	2	20.0%	5	45.5%	
	3	2	20.0%	5	45.5%	
	4	2	20.0%	0	0.0%	
	5	1	10.0%	0	0.0%	
Place of taking lunch	At Work	1	10.0%	1	9.1%	0.5
	House	7	70.0%	9	81.8%	
	House, at Work	1	10.0%	0	0.0%	
	House, Restaurants	1	10.0%	0	0.0%	
	Restaurant	0	0.0%	1	9.1%	
Do you engage in regular exercise or physical activity?	No	6	60.0%	4	36.4%	0.2
	Yes	4	40.0%	7	63.6%	
Physical activity, how much per week?	2-3 times a week	1	10.0%	1	9.1%	0.4
	Daily	0	0.0%	2	18.2%	
	None	6	60.0%	4	36.4%	
	Once a week	1	10.0%	0	0.0%	

	Once or twice a week	0	0.0%	1	9.1%	
	Several times a week	2	20.0%	3	27.3%	
Do you follow a special diet during the flare of the disease?	No	4	40.0%	5	45.5%	0.8
	Yes	6	60.0%	6	54.5%	
If yes, which of the following do you follow?	By avoiding fried food and foods with sauce	2	20.0%	0	0.0%	0.3
	By avoiding raw meat, etc.	1	10.0%	0	0.0%	
	Excluding fruits and vegetables	0	0.0%	1	9.1%	
	Excluding whole fruits and vegetables, by avoiding fried foods and foods with sauce	0	0.0%	1	9.1%	
	Excluding whole fruits and vegetables, by avoiding fried foods and foods with sauce, no fat, no added Sugar	0	0.0%	1	9.1%	
	Excluding whole fruit and vegetables, by eating vegetables and fruit cooked and peeled only, by avoiding fried foods and foods with sauce	0	0.0%	1	9.1%	
	Excluding garlic, onions, beans	1	10.0%	0	0.0%	
	Excluding green leafy veggies (spinach, etc.), apples, pulses	1	10.0%	0	0.0%	
	Excluding green onion, spices, tomato paste	1	10.0%	0	0.0%	
	Excluding only chili pepper	0	0.0%	1	9.1%	
	Excluding pulses	0	0.0%	1	9.1%	
	No specific diet followed	4	40.0%	5	45.5%	
If yes, duration of this diet	2 weeks	0	0.0%	2	18.2%	0.3
	24 hours	1	10.0%	0	0.0%	
	3 days	1	10.0%	0	0.0%	
	No specific diet	4	40.0%	5	45.5%	
	Since being diagnosed with CD	4	40.0%	3	27.3%	
	Weeks	0	0.0%	1	9.1%	
This diet advised by whom?	Dietitian	0	0.0%	1	9.1%	0.6
	Gastroenterologist	2	20.0%	1	9.1%	
	Media	0	0.0%	1	9.1%	
	No specific diet followed	4	40.0%	5	45.5%	
	Personal Belief	4	40.0%	3	27.3%	
Do you follow a diet out of the flare of the disease	No	6	60.0%	8	72.7%	0.5
	Yes	4	40.0%	3	27.3%	
If yes, which of the following do you follow?	By avoiding fried food and foods with sauce	1	10.0%	0	0.0%	0.4
	Excluding whole fruits and vegetables, by avoiding fried foods and foods with sauce	0	0.0%	1	9.1%	
	Excluding garlic, onions, beans	1	10.0%	0	0.0%	
	Excluding green leafy veggies (spinach, etc.), apples, pulses	1	10.0%	0	0.0%	

	Excluding green onion, spices, tomato paste	1	10.0%	0	0.0%	
	Excluding only chili pepper	0	0.0%	1	9.1%	
	Excluding pulses	0	0.0%	1	9.1%	
	No specific diet followed	6	60.0%	8	72.7%	
If yes, duration of this diet	No specific diet followed	6	60.0%	8	72.7%	0.5
	Since being diagnosed with CD	4	40.0%	3	27.3%	
Source of diet	No specific diet followed	6	60.0%	8	72.7%	0.5
	Personal Belief	4	40.0%	3	27.3%	
Do you follow-up with a nutrition specialist?	No	10	100.0%	11	100.0%	-
If yes, with whom do you do nutritional follow-up?	Not applicable	10	100.0%	11	100.0%	-
What type of water do you consume on a regular basis?	Filtered Gallons	3	30.0%	2	18.2%	0.5
	Water Bottle	7	70.0%	9	81.8%	
Do you consume soft drinks/soda?	No	4	40.0%	5	45.5%	0.8
	Yes	6	60.0%	6	54.5%	
Which type of soft drinks/soda do you drink?	No sugar / sweetened (Cola light, Cola Zero ....)	2	20.0%	0	0.0%	0.2
	Not applicable, I do not consume any soft drinks/soda	4	40.0%	5	45.5%	
	Sweetened	3	30.0%	6	54.5%	
	Sweetened, no sugar / sweetened (Cola light, Cola Zero ....)	1	10.0%	0	0.0%	

Table S2. Dietary Supplements use by patients with CD per study groups at baseline level.

		Study Group				
		Control		Intervention		
		Count	Column N %	Count	Column N %	p-value
Supplements	No	6	60.0%	4	36.4%	0.2
	Yes	4	40.0%	7	63.6%	
Type	Magnesium	0	0.0%	1	9.1%	0.4
	Magnesium, Vitamin B9	1	10.0%	0	0.0%	
	Multi-vitamins	1	10.0%	0	0.0%	
	Multi-vitamins, B – Complex supplement	0	0.0%	1	9.1%	
	Multi-vitamins, Magnesium	0	0.0%	1	9.1%	
	Not applicable	6	60.0%	4	36.4%	
	Vitamin B9	1	10.0%	0	0.0%	
	Vitamin C, Magnesium	0	0.0%	1	9.1%	
	Vitamin C, Vitamin D	0	0.0%	1	9.1%	
	Vitamin C, Vitamin D, Magnesium	0	0.0%	1	9.1%	
	Vitamin C, Vitamin D, Omega-3	0	0.0%	1	9.1%	
	Vitamin D, Vitamin E, Vitamin B12	1	10.0%	0	0.0%	
Multi-vitamins	Never	9	90.0%	9	81.8%	0.2
	Once a week	0	0.0%	2	18.2%	
	Several times a week	1	10.0%	0	0.0%	
Antioxidants	Never	9	90.0%	11	100.0%	0.2

	Several times a week	1	10.0%	0	0.0%	
Vitamin A	Never	9	90.0%	11	100.0%	0.2
	Several times a week	1	10.0%	0	0.0%	
Vitamin C	Daily basis	1	10.0%	1	9.1%	0.2
	Never	9	90.0%	7	63.6%	
	Once a month	0	0.0%	3	27.3%	
Vitamin D	Daily basis	1	10.0%	0	0.0%	0.3
	Never	8	80.0%	8	72.7%	
	Once a week	1	10.0%	3	27.3%	
Vitamin E	Daily basis	1	10.0%	0	0.0%	0.2
	Never	8	80.0%	11	100.0%	
	Several times a week	1	10.0%	0	0.0%	
Beta- Carotene	Never	10	100.0%	11	100.0%	-
B – Complex	Never	9	90.0%	10	90.9%	0.3
	Once a week	0	0.0%	1	9.1%	
	Several times a week	1	10.0%	0	0.0%	
B2 Riboflavin	Never	9	90.0%	11	100.0%	0.2
	Several times a week	1	10.0%	0	0.0%	
B5 Pantothenic	Never	9	90.0%	11	100.0%	0.2
	Several times a week	1	10.0%	0	0.0%	
B6 Pyridoxine	Never	9	90.0%	11	100.0%	0.2
	Several times a week	1	10.0%	0	0.0%	
Vitamin B12	Never	8	80.0%	11	100.0%	0.2
	Once a week	1	10.0%	0	0.0%	
	Several times a week	1	10.0%	0	0.0%	
Calcium	Never	9	90.0%	11	100.0%	0.2
	Several times a week	1	10.0%	0	0.0%	
Chromium	Never	9	90.0%	11	100.0%	0.2
	Several times a week	1	10.0%	0	0.0%	
Iron	Daily basis	1	10.0%	0	0.0%	0.2
	Never	9	90.0%	11	100.0%	
Zinc	Never	9	90.0%	11	100.0%	0.3
	Several times a week	1	10.0%	0	0.0%	
Magnesium	Daily basis	0	0.0%	1	9.1%	0.2
	Never	9	90.0%	7	63.6%	
	Once a week	0	0.0%	2	18.2%	
	Several times a week	1	10.0%	1	9.1%	
Potassium	Never	9	90.0%	11	100.0%	0.2
	Several times a week	1	10.0%	0	0.0%	
Phosphorus	Never	9	90.0%	11	100.0%	0.1
	Several times a week	1	10.0%	0	0.0%	
Vitamin B9	Daily basis	2	20.0%	0	0.0%	0.1
	Never	7	70.0%	11	100.0%	
	Several times a week	1	10.0%	0	0.0%	
Selenium	Never	9	90.0%	11	100.0%	0.2
	Several times a week	1	10.0%	0	0.0%	
Omega-3	Daily basis	0	0.0%	1	9.1%	0.3
	Never	10	100.0%	10	90.9%	
Copper	Never	10	100.0%	11	100.0%	-
Source of prescription	Gastroenterologist	0	0.0%	2	18.2%	0.1
	Medical doctor	4	40.0%	2	18.2%	
	Not applicable, I do not take any supplements	6	60.0%	4	36.4%	
	Personal Belief	0	0.0%	3	27.3%	

Table S3. Comparison between nutrients profiles of both study groups at baseline.

	Group	N	Mean	Std. Deviation	p-value
Energy	Control	10	1914.56	1006.94	0.09
	Intervention	10	2643.91	1920.35	
Protein	Control	10	61.17	21.17	0.001*
	Intervention	10	98.13	66.73	
CHO	Control	10	224.65	135.33	0.3
	Intervention	10	294.83	170.26	
Fat	Control	10	85.68	52.65	0.1
	Intervention	10	121.28	110.35	
Cholesterol	Control	10	203.51	217.58	0.2
	Intervention	10	347.20	357.80	
Saturated Fatty Acid	Control	10	16.53	7.705	0.09
	Intervention	10	26.92	26.72	
Monounsaturated fatty acid	Control	10	30.97	25.69	0.08
	Intervention	10	43.97	49.81	
Poly Unsaturated Fatty Acid	Control	10	19.03	17.13	0.1
	Intervention	10	24.70	25.62	
Oleic acid	Control	10	29.08	24.79	0.07
	Intervention	10	41.73	48.39	
Linoleic acid	Control	10	17.57	16.11	0.1
	Intervention	10	22.40	23.53	
Linolenic acid	Control	10	1.19	1.12	0.06
	Intervention	10	1.68	1.89	
EPA	Control	10	.00	.000	0.001*
	Intervention	10	.02	.04	
DHA	Control	10	.02	.04	0.01*
	Intervention	10	.07	.12	
Trans fatty acid	Control	9	.41	.54	0.7
	Intervention	10	.75	.51	
Sodium	Control	10	2479.51	1094.78	0.01*
	Intervention	10	4599.67	3903.04	
Potassium	Control	10	2422.27	1720.74	0.1
	Intervention	10	3246.41	2752.77	
Beta carotene	Control	10	677.22	959.40	0.2
	Intervention	10	1662.23	1207.70	
Alpha carotene	Control	10	195.18	325.44	0.1
	Intervention	10	529.92	489.39	
Lutein	Control	10	559.59	509.40	0.3
	Intervention	10	1032.61	640.35	
Lycopene	Control	10	2691.00	6662.84	0.2
	Intervention	10	4679.20	8612.42	
Vitamin C	Control	10	40.29	36.18	0.6
	Intervention	10	55.70	39.74	
Calcium	Control	10	272.85	96.77	0.03*
	Intervention	10	528.74	483.54	
Iron	Control	10	10.37	4.34	0.01*
	Intervention	10	13.78	9.64	
Vitamin D	Control	10	28.11	52.84	0.008*
	Intervention	10	1.38	2.51	
Vitamin E	Control	10	5.97	5.47	0.2
	Intervention	10	7.94	6.93	
Thiamin	Control	10	1.05	.60	0.06
	Intervention	10	1.33	.98	
Riboflavin	Control	10	.92	.43	0.04*
	Intervention	10	1.17	.78	
Niacin	Control	10	16.19	8.81	0.01*

	Intervention	10	22.61	19.39	
Pyridoxine	Control	10	1.47	1.17	0.08
	Intervention	10	2.20	2.08	
Folate	Control	10	255.46	123.15	0.1
	Intervention	10	324.09	191.36	
Vitamin B12	Control	10	2.05	1.77	0.5
	Intervention	10	2.31	2.37	
Biotin	Control	10	11.71	14.74	0.7
	Intervention	10	13.37	16.25	
Vitamin B5	Control	10	3.74	2.32	0.1
	Intervention	10	4.56	4.06	
Vitamin K	Control	10	50.57	37.79	0.06
	Intervention	10	112.74	136.38	
Phosphorus	Control	10	879.07	486.30	0.06
	Intervention	10	1122.66	881.12	
Magnesium	Control	10	174.33	110.77	0.07
	Intervention	10	243.35	195.51	
Zinc	Control	10	7.59	3.59	0.02*
	Intervention	10	9.12	6.97	
Copper	Control	10	.93	.55	0.06
	Intervention	10	1.19	.91	
Manganese	Control	10	1.53	.82	0.1
	Intervention	10	1.84	1.31	
Selenium	Control	10	64.13	32.32	0.01*
	Intervention	10	82.41	68.94	
Fluoride	Control	10	437.46	660.20	0.1
	Intervention	10	280.40	519.62	
Chromium	Control	8	.01	.03	0.03*
	Intervention	8	.00	.00	
Dietary fiber	Control	10	15.21	11.50	0.3
	Intervention	11	22.72	14.62	
Soluble fiber	Control	10	.01	.03	0.02*
	Intervention	11	.05	.10	
Insoluble fiber	Control	10	.05	.10	0.01*
	Intervention	11	.27	.46	
Crude fiber	Control	10	1.30	1.20	0.8
	Intervention	11	1.52	1.32	
Sugar	Control	10	35.36	34.46	0.9
	Intervention	11	40.33	29.18	
Glucose	Control	10	4.13	3.66	0.9
	Intervention	10	6.72	7.35	
Galactose	Control	10	.26	.34	0.8
	Intervention	11	.23	.33	
Fructose	Control	10	4.94	5.92	0.2
	Intervention	10	8.44	10.14	
Sucrose	Control	10	3.53	3.01	0.1
	Intervention	11	5.45	4.28	
Lactose	Control	10	1.48	1.59	0.3
	Intervention	11	1.95	4.65	
Tryptophan	Control	10	400.42	294.13	0.1
	Intervention	11	508.45	533.08	
Threonine	Control	10	1329.42	1083.14	0.2
	Intervention	11	1707.01	1892.12	
Isoleucine	Control	10	1506.46	1280.37	0.1
	Intervention	11	1959.77	2204.52	
Leucine	Control	10	2541.94	2114.44	0.2

	Intervention	11	3200.97	3506.20	
Lysine	Control	10	2346.52	2135.15	0.2
	Intervention	11	3033.20	3687.39	
Methionine	Control	10	768.63	698.39	0.1
	Intervention	11	998.97	1202.54	
Cystine	Control	10	479.60	394.99	0.2
	Intervention	11	626.05	642.75	
Phenyl alanine	Control	10	1637.86	1122.00	0.1
	Intervention	11	2138.35	2161.85	
Tyrosine	Control	10	1099.60	893.01	0.1
	Intervention	11	1390.65	1554.22	
Valine	Control	10	1818.68	1380.51	0.1
	Intervention	11	2323.43	2491.02	
Arginine	Control	10	2089.01	1632.49	0.1
	Intervention	11	2595.11	2733.25	0.2
Histidine	Control	10	896.72	788.28	
	Intervention	11	1119.36	1253.71	0.3
Alanine	Control	10	1716.04	1585.23	
	Intervention	11	2099.22	2444.05	0.1
Aspartic acid	Control	10	4141.55	2793.65	
	Intervention	11	5450.93	5730.99	
Glutamic acid	Control	10	6412.77	4275.60	0.1
	Intervention	11	8049.67	7350.20	
Glycine	Control	10	1657.19	1592.03	0.5
	Intervention	11	1848.10	2074.34	
Proline	Control	10	1931.51	1415.16	0.2
	Intervention	11	2279.19	2085.84	
Serine	Control	10	1552.13	1191.47	0.2
	Intervention	11	1964.01	1999.89	
Caffeine	Control	10	68.09	114.33	0.04*
	Intervention	11	34.52	39.25	

Table S4. QALYS between the two study groups after 2 months supplementations by probiotics.

	Group	N	Mean	Std. Deviation	p-value
Raw Score for Physical Health	1	10	24.10	3.84274	0.7
	2	11	25.81	3.06001	
Transformed Physical health (0-100)	1	10	61.40	12.929	0.8
	2	11	67.82	11.116	
Raw score for Psychology	1	10	22.90	3.281	0.2
	2	11	22.00	2.720	
Transformed Psychology (0-100)	1	10	70.50	12.774	0.2
	2	11	66.55	10.885	
Raw Score for social relationships	1	10	11.20	2.044	0.7
	2	11	11.55	2.067	
Transformed social relationships (0-100)	1	10	68.80	17.492	0.6
	2	11	70.36	17.761	
Raw environment	1	10	25.00	3.018	0.6
	2	11	27.36	3.529	
Transformed score (0-100) for Environment	1	10	54.40	9.732	0.8
	2	11	62.18	11.161	

Table S5. The nutrition status and dietary patterns of the two study groups after two months supplementation.

		Group				
		control		intervention		p-value
		N	N %	N	N %	
SGA	Moderately malnourished	2	20.0%	3	27.3%	0.9
	Well nourished	8	80.0%	8	72.7%	
Are your daily meals regular?	No	5	50.0%	3	27.3%	0.6
	Yes	5	50.0%	8	72.7%	
Did your food intake change in the past 2 months?	Food intake remained the same	1	10.0%	4	36.4%	0.4
	Intake decreased	5	50.0%	1	9.1%	
	Intake increased	4	40.0%	6	54.5%	
Nb meals day	2	5	50.0%	5	45.5%	0.2
	3	3	30.0%	5	45.5%	
	4	2	20.0%	1	9.1%	
Do you engage in regular exercise or physical activity?	No	5	50.0%	6	54.5%	0.4
	Yes	5	50.0%	5	45.5%	
If he/she performs sports, how much per week?	2-3 times a week	2	20.0%	2	18.2%	0.1
	Not applicable, I do not perform any physical activity	5	50.0%	6	54.5%	
	Once a week	1	10.0%	1	9.1%	
	Several times a week	2	20.0%	2	18.2%	
Do you follow a special diet during the "flare" of the disease?	No	2	20.0%	2	18.2%	0.9
	Yes	8	80.0%	9	81.8%	
Type diet during flares	Avoiding spices and sauces	1	10.0%	0	0.0%	0.8
	By avoiding fried food and foods with sauce	2	20.0%	1	9.1%	
	By avoiding fried foods and foods with sauce, by consuming oral nutritional supplements, No added salt, No added sugar	1	10.0%	0	0.0%	
	By avoiding fried foods and foods with sauce, Elimination of spices, garlic, etc.	0	0.0%	1	9.1%	
	By avoiding fried foods and foods with sauce, excluding pulses, excluding fruits and vegetables	1	10.0%	0	0.0%	
	Elimination of green onions, spices, garlic, tomato paste	0	0.0%	1	9.1%	
	Elimination of raw meat and raw foods	0	0.0%	1	9.1%	
	Exclude green leafy vegetables, excluding pulses	1	10.0%	0	0.0%	
	Excluding whole fruits and vegetables, by avoiding fried foods and foods with sauce	0	0.0%	1	9.1%	
	Excluding fruits and vegetables, by avoiding fried foods and foods with sauce, Excluding caffeinated beverages	0	0.0%	1	9.1%	
	Excluding fruits and vegetables, by eating vegetables and fruits cooked and peeled only, by avoiding fried foods and foods with sauce	1	10.0%	1	9.1%	

	Excluding garlic, onions, oat, humus, beans, lentils, chili pepper	0	0.0%	1	9.1%	
	Excluding only chili pepper	1	10.0%	0	0.0%	
	Excluding pulses	0	0.0%	1	9.1%	
	No specific diet followed	2	20.0%	2	18.2%	
Duration of diet during flare	1 day	2	20.0%	1	9.1%	0.7
	1-2 days	1	10.0%	0	0.0%	
	2-3 days	1	10.0%	2	18.2%	
	4-5 days	0	0.0%	2	18.2%	
	Depending on the length of flare	1	10.0%	1	9.1%	
	No specific diet followed	2	20.0%	2	18.2%	
	Since I was diagnosed with IBD	3	30.0%	3	27.3%	
Source of diet during flare	Dietitian	0	0.0%	1	9.1%	0.4
	Gastroenterologist	3	30.0%	1	9.1%	
	No specific diet followed	2	20.0%	2	18.2%	
	Personal Belief	5	50.0%	7	63.6%	
Diet out of flare	No	5	50.0%	7	63.6%	0.09
	Yes	5	50.0%	4	36.4%	
Type diet out of flare	By avoiding fried food and foods with sauce	1	10.0%	0	0.0%	
	By avoiding fried foods and foods with sauce, by consuming oral nutritional supplements, no added salt, no added sugar	1	10.0%	0	0.0%	
	Elimination of green onions, spices, garlic, tomato paste	0	0.0%	1	9.1%	
	Elimination of raw meat and raw foods	0	0.0%	1	9.1%	
	Exclude green leafy vegetables, excluding pulses	1	10.0%	0	0.0%	
	Excluding garlic, onions, oat, humus, beans, lentils, chili pepper	0	0.0%	1	9.1%	
	Excluding only chili pepper	1	10.0%	0	0.0%	
	Excluding tomatoes (fresh and cooked), excluding pulses	0	0.0%	1	9.1%	
	No added sugar, by avoiding fried foods and foods with sauce, excluding fruits and vegetables	1	10.0%	0	0.0%	
	No specific diet followed	5	50.0%	7	63.6%	
Duration of diet out of flare	2 months	0	0.0%	1	9.1%	0.7
	2 years	1	10.0%	0	0.0%	
	4 years	1	10.0%	0	0.0%	
	No specific diet followed	5	50.0%	7	63.6%	
	Since I was diagnosed with IBD	3	30.0%	3	27.3%	
Source of diet out of flare	Gastroenterologist	1	10.0%	1	9.1%	0.2
	No specific diet followed	5	50.0%	7	63.6%	
	Personal Belief	4	40.0%	3	27.3%	
Current supplement	No	6	60.0%	3	27.3%	0.7
	Yes	4	40.0%	8	72.7%	
Type of supplement	Magnesium	0	0.0%	2	18.2%	0.5
	Magnesium, Vitamin B9	0	0.0%	1	9.1%	
	Multi-vitamins	0	0.0%	1	9.1%	
	Not applicable	6	60.0%	3	27.3%	
	Vitamin A	0	0.0%	1	9.1%	

	Vitamin B12	1	10.0%	0	0.0%	
	Vitamin B9	0	0.0%	1	9.1%	
	Vitamin C, B2 Riboflavin, B6 Pyridoxine, Vitamin B12, Iron, Vitamin B9, Copper	1	10.0%	0	0.0%	
	Vitamin C, Vitamin D, Magnesium	1	10.0%	0	0.0%	
	Vitamin C, Vitamin D, Zinc, Omega-3	0	0.0%	1	9.1%	
	Vitamin D, B – Complex supplement, Vitamin B9	0	0.0%	1	9.1%	
	Vitamin D, Iron, Magnesium	1	10.0%	0	0.0%	

Table S6. Anthropometric variation among each group after two months supplementation.

	Group	N	Mean	Std. Deviation	p-value
Waist circumference	Control	10	90.30	17.651	0.8
	Intervention	11	84.27	15.533	
Weight	Control	10	65.91	13.498	0.6
	Intervention	11	78.06	11.998	
Body mass index	Control	10	23.52	3.384	0.3
	Intervention	11	28.08	3.331	
Total body water	Control	10	35.28	8.216	0.1
	Intervention	11	38.48	8.643	
Intracellular water	Control	10	21.78	5.212	0.7
	Intervention	11	23.85	5.575	
Extra cellular water	Control	10	13.50	3.013	0.6
	Intervention	11	14.63	3.084	
Protein	Control	10	9.40	2.244	0.8
	Intervention	11	10.33	2.405	
Minerals	Control	10	3.41	.716	0.7
	Intervention	11	3.65	.799	
Body Fat Mass	Control	10	17.83	8.570	0.6
	Intervention	11	25.61	8.508	
Soft Lean Mass	Control	10	45.28	10.605	0.4
	Intervention	11	49.44	11.187	
Free Fat Mass	Control	10	48.08	11.156	0.1
	Intervention	11	52.45	11.825	
Skeletal Muscle Mass	Control	10	26.41	6.835	0.7
	Intervention	11	29.13	7.270	
Percent Body Fat	Control	10	26.83	11.117	0.6
	Intervention	11	32.87	10.875	
Basal Metabolic Rate	Control	10	1407.90	241.224	0.2
	Intervention	11	1502.91	255.381	
Waist Hip Ratio	Control	10	.89	.073	0.4
	Intervention	11	.97	.083	
AMC	Control	10	26.20	2.930	0.1
	Intervention	11	29.36	2.609	

Table S7. Anthropometric variation between groups after two months supplementation.

ANOVA						
		Sum of Squares	df	Mean Square	F	Sig.
WC_T1	Between Groups	190.290	1	190.290	.693	.415
	Within Groups	5216.782	19	274.567		
	Total	5407.071	20			
H	Between Groups	.260	1	.260	.002	.961
	Within Groups	2047.407	19	107.758		
	Total	2047.667	20			
Weight_T1	Between Groups	773.724	1	773.724	4.774	.042
	Within Groups	3079.274	19	162.067		
	Total	3852.998	20			
BMI_T1	Between Groups	109.006	1	109.006	9.678	.006
	Within Groups	213.992	19	11.263		
	Total	322.998	20			
TBW_T1	Between Groups	53.699	1	53.699	.753	.396
	Within Groups	1354.472	19	71.288		
	Total	1408.171	20			
ICW_T1	Between Groups	22.543	1	22.543	.771	.391
	Within Groups	555.303	19	29.226		
	Total	577.847	20			
ECW_T1	Between Groups	6.656	1	6.656	.715	.408
	Within Groups	176.762	19	9.303		
	Total	183.418	20			
Protein_T1	Between Groups	4.504	1	4.504	.830	.374
	Within Groups	103.162	19	5.430		
	Total	107.666	20			
minerals_T1	Between Groups	.305	1	.305	.527	.477
	Within Groups	11.000	19	.579		
	Total	11.305	20			
BFM_T1	Between Groups	316.979	1	316.979	4.348	.049
	Within Groups	1384.990	19	72.894		
	Total	1701.970	20			
SLM_T1	Between Groups	90.490	1	90.490	.760	.394

	Within Groups	2263.641	19	119.139		
	Total	2354.131	20			
FFM_T1	Between Groups	100.240	1	100.240	.756	.395
	Within Groups	2518.323	19	132.543		
	Total	2618.563	20			
SMM_T1	Between Groups	38.676	1	38.676	.774	.390
	Within Groups	949.051	19	49.950		
	Total	987.727	20			
PBF_T1	Between Groups	191.267	1	191.267	1.583	.224
	Within Groups	2294.963	19	120.788		
	Total	2486.230	20			
BMR_T1	Between Groups	47282.858	1	47282.858	.764	.393
	Within Groups	1175893.809	19	61889.148		
	Total	1223176.667	20			
WHR_T1	Between Groups	.026	1	.026	4.254	.053
	Within Groups	.116	19	.006		
	Total	.142	20			
AMC_T1	Between Groups	52.426	1	52.426	6.852	.017
	Within Groups	145.365	19	7.651		
	Total	197.791	20			

Table S8. Dietary patterns, malnutrition and lifestyle assessment among both groups each one alone after two months of supplementation.

		Control			Intervention		
		Count	Column N %	p-value	Count	Column N %	p-value
SGA at T0	Mild/moderate undernutrition	2	20.0%	0.7	5	45.5%	0.2
	Severe undernutrition	1	10.0%		0	0.0%	
	Well nourished	7	70.0%		6	54.5%	
SGA at T1	Moderately malnourished	2	20.0%	0.1	3	27.3%	0.3
	Well nourished	8	80.0%		8	72.7%	
Are your daily meals regular?	No	4	40.0%	0.9	7	63.6%	0.8
	Yes	6	60.0%		4	36.4%	
Are your daily meals regular?	No	5	50.0%		3	27.3%	
	Yes	5	50.0%		8	72.7%	
	Food intake remained the same	1	10.0%	0.7	4	36.4%	0.9

Did your food intake change in the past 2 months?	Intake decreased	5	50.0%		1	9.1%	
	Intake increased	4	40.0%		6	54.5%	
Number of meals per day	1	3	30.0%	0.5	1	9.1%	0.7
	2	2	20.0%		5	45.5%	
	3	2	20.0%		5	45.5%	
	4	2	20.0%		0	0.0%	
	5	1	10.0%		0	0.0%	
Number of meals per day at T1	2	5	50.0%	0.1	5	45.5%	0.6
	3	3	30.0%		5	45.5%	
	4	2	20.0%		1	9.1%	
Do you follow a special diet during the flare of the disease?	No	4	40.0%	0.4	5	45.5%	0.1
	Yes	6	60.0%		6	54.5%	
If yes, which of the following do you follow?	By avoiding fried food and foods with sauce	2	20.0%	0.9	0	0.0%	0.4
	By avoiding raw meat, etc.	1	10.0%		0	0.0%	
	Excluding whole fruits and vegetables	0	0.0%		1	9.1%	
	Excluding whole fruits and vegetables, by avoiding fried foods and foods with sauce	0	0.0%		1	9.1%	
	Excluding whole fruits and vegetables, by avoiding fried foods and foods with sauce, No fat, No added Sugar	0	0.0%		1	9.1%	
	Excluding whole fruits and vegetables, by eating vegetables and fruits cooked and peeled only, by avoiding fried foods and foods with sauce	0	0.0%		1	9.1%	
	Excluding garlic, onions, beans	1	10.0%		0	0.0%	
	Excluding green leafy veggies (spinach, etc.), apples, pulses	1	10.0%		0	0.0%	
	Excluding green onion, spices, tomato paste	1	10.0%		0	0.0%	
	Excluding only chili pepper	0	0.0%		1	9.1%	
	Excluding pulses	0	0.0%		1	9.1%	
	No specific diet followed	4	40.0%		5	45.5%	
If yes, duration of this diet	2 weeks	0	0.0%	0.1	2	18.2%	0.2

	24 hours	1	10.0%		0	0.0%	
	3 days	1	10.0%		0	0.0%	
	No specific diet followed	4	40.0%		5	45.5%	
	Since I was diagnosed with IBD	4	40.0%		3	27.3%	
	Weeks	0	0.0%		1	9.1%	
This diet advised by whom?	Dietitian	0	0.0%	0.9	1	9.1%	0.7
	Gastroenterologist	2	20.0%		1	9.1%	
	Media	0	0.0%		1	9.1%	
	No specific diet followed	4	40.0%		5	45.5%	
	Personal Belief	4	40.0%		3	27.3%	
Do you follow a diet out of the flare of the disease	No	6	60.0%	0.2	8	72.7%	0.6
	Yes	4	40.0%		3	27.3%	
If yes, which of the following do you follow?	By avoiding fried food and foods with sauce	1	10.0%	0.8	0	0.0%	0.1
	Excluding whole fruits and vegetables, by avoiding fried foods and foods with sauce	0	0.0%		1	9.1%	
	Excluding garlic, onions, beans	1	10.0%		0	0.0%	
	Excluding green leafy veggies (spinach, etc.), apples, pulses	1	10.0%		0	0.0%	
	Excluding green onion, spices, tomato paste	1	10.0%		0	0.0%	
	Excluding only chili pepper	0	0.0%		1	9.1%	
	Excluding pulses	0	0.0%		1	9.1%	
	No specific diet followed	6	60.0%		8	72.7%	
If yes, duration of this diet	No specific diet followed	6	60.0%	0.9	8	72.7%	0.6
	Since I was diagnosed with IBD	4	40.0%		3	27.3%	
This diet advised by whom?	No specific diet followed	6	60.0%		8	72.7%	
	Personal Belief	4	40.0%		3	27.3%	
Do you follow a special diet during the flare of the disease?	No	2	20.0%	0.5	2	18.2%	0.1
	Yes	8	80.0%		9	81.8%	
Type of diet during flare at T1	Avoiding spices and sauces	1	10.0%	0.6	0	0.0%	0.8
	By avoiding fried food and foods with sauce	2	20.0%		1	9.1%	
	By avoiding fried foods and foods with sauce, by consuming oral nutritional supplements, No added salt, No added sugar	1	10.0%		0	0.0%	

	By avoiding fried foods and foods with sauce, Elimination of spices, garlic, etc.	0	0.0%		1	9.1%	
	By avoiding fried foods and foods with sauce, excluding pulses, excluding whole fruits and vegetables	1	10.0%		0	0.0%	
	Elimination of green onions, spices, garlic, tomato paste	0	0.0%		1	9.1%	
	Elimination of raw meat and raw foods	0	0.0%		1	9.1%	
	Exclude green leafy vegetables, Excluding pulses	1	10.0%		0	0.0%	
	Excluding whole fruits and vegetables, by avoiding fried foods and foods with sauce	0	0.0%		1	9.1%	
	Excluding whole fruits and vegetables, by avoiding fried foods and foods with sauce, excluding caffeinated beverages	0	0.0%		1	9.1%	
	Excluding whole fruits and vegetables, by eating vegetables and fruits cooked and peeled only, by avoiding fried foods and foods with sauce	1	10.0%		1	9.1%	
	Excluding garlic, onions, oat, humus, beans, lentils, chili pepper	0	0.0%		1	9.1%	
	Excluding only chili pepper	1	10.0%		0	0.0%	
	Excluding pulses	0	0.0%		1	9.1%	
	No specific diet followed	2	20.0%		2	18.2%	
Duration of diet during flare at T1	1 day	2	20.0%	0.6	1	9.1%	0.7
	1-2 days	1	10.0%		0	0.0%	
	2-3 days	1	10.0%		2	18.2%	
	4-5 days	0	0.0%		2	18.2%	
	Depending on the length of flare	1	10.0%		1	9.1%	
	No specific diet followed	2	20.0%		2	18.2%	
	Since I was diagnosed with IBD	3	30.0%		3	27.3%	
	Dietitian	0	0.0%	0.9	1	9.1%	0.1

Source of diet during flare at T1	Gastroenterologist	3	30.0%		1	9.1%	
	No specific diet followed	2	20.0%		2	18.2%	
	Personal Belief	5	50.0%		7	63.6%	
Diet out of flare at T1	No	5	50.0%	0.4	7	63.6%	0.6
	Yes	5	50.0%		4	36.4%	
Type of diet out of flareT1	By avoiding fried food and foods with sauce	1	10.0%	0.1	0	0.0%	0.4
	By avoiding fried foods and foods with sauce, by consuming oral nutritional supplements, No added salt, No added sugar	1	10.0%		0	0.0%	
	Elimination of green onions, spices, garlic, tomato paste	0	0.0%		1	9.1%	
	Elimination of raw meat and raw foods	0	0.0%		1	9.1%	
	Exclude green leafy vegetables, excluding pulses	1	10.0%		0	0.0%	
	Excluding garlic, onions, oat, humus, beans, lentils, chili pepper	0	0.0%		1	9.1%	
	Excluding only chili pepper	1	10.0%		0	0.0%	
	Excluding tomatoes (fresh and cooked), excluding pulses	0	0.0%		1	9.1%	
	No added sugar, by avoiding fried foods and foods with sauce, excluding whole fruits and vegetables	1	10.0%		0	0.0%	
	No specific diet followed	5	50.0%		7	63.6%	
Duration of diet out of flare T1	2 months	0	0.0%	0.9	1	9.1%	0.7
	2 years	1	10.0%		0	0.0%	
	4 years	1	10.0%		0	0.0%	
	No specific diet followed	5	50.0%		7	63.6%	
	Since I was diagnosed with IBD	3	30.0%		3	27.3%	
Source of diet at T1	Gastroenterologist	1	10.0%	0.2	1	9.1%	0.4
	No specific diet followed	5	50.0%		7	63.6%	
	Personal belief	4	40.0%		3	27.3%	

Table S9. Comparisons between study groups regarding nutrients intake after two months of the study.

	Group	N	Mean	Std. Deviation	p-value

Energy	Control	10	3583.20	2711.510	0.9
	Intervention	11	3999.84	2554.270	
Protein	Control	10	106.24	83.701	0.3
	Intervention	11	151.70	131.751	
CHO	Control	10	428.81	334.805	0.3
	Intervention	11	444.45	235.470	
Fat	Control	10	165.29	143.726	0.9
	Intervention	11	182.12	135.748	
Cholesterol	Control	10	260.87	272.914	0.1
	Intervention	11	474.52	466.494	
Saturated fatty acid	Control	10	33.80	27.722	0.8
	Intervention	11	38.44	24.725	
Monounsaturated fatty acid	Control	10	67.55	67.535	0.8
	Intervention	11	69.89	61.486	
Polyunsaturated fatty acid	Control	10	33.44	40.844	0.8
	Intervention	11	38.31	35.608	
Oleic	Control	10	63.86	64.716	0.9
	Intervention	11	65.96	59.006	
Linoleic	Control	10	30.63	37.577	0.5
	Intervention	11	34.69	32.896	
Linolenic	Control	10	2.35	2.884	0.6
	Intervention	11	2.50	2.458	
EPA	Control	10	.03	.047	0.3
	Intervention	11	.03	.041	
DHA	Control	10	.07	.107	02
	Intervention	11	.10	.135	
Trans fatty acid	Control	10	2.26	4.175	0.6
	Intervention	11	1.43	1.631	
Sodium	Control	10	3205.93	2564.807	0.8
	Intervention	11	4465.27	2735.368	
Potassium	Control	10	5265.98	4575.630	0.2
	Intervention	11	5243.18	3891.483	
Lycopene	Control	10	4939.29	9871.182	0.2
	Intervention	11	3932.75	3802.365	
Vitamin C	Control	10	74.89	58.400	0.8
	Intervention	11	79.48	42.479	
Calcium	Control	10	567.13	200.172	0.01*
	Intervention	11	1028.76	645.704	
Iron	Control	10	17.60	12.491	0.6
	Intervention	11	21.24	12.458	
Vitamin D	Control	10	1.41	1.379	0.3
	Intervention	11	2.03	3.197	
Vitamin E	Control	9	2.11	2.628	0.5

	Intervention	11	2.41	1.684	
Alpha tocopherol	Control	10	10.00	7.757	0.5
	Intervention	11	12.96	9.257	
Thiamin	Control	10	1.75	1.368	0.6
	Intervention	11	1.96	1.153	
Riboflavin	Control	10	1.45	.603	0.04*
	Intervention	11	2.03	1.229	
Niacin	Control	10	29.57	21.950	0.2
	Intervention	11	44.00	53.262	
Pyridoxine	Control	10	3.50	3.096	0.7
	Intervention	11	3.81	3.606	
Folate	Control	10	459.90	313.347	0.013*
	Intervention	11	564.73	186.683	
Cobalamin	Control	10	5.16	9.575	0.5
	Intervention	11	8.39	16.404	
Biotin	Control	10	12.93	12.346	0.1
	Intervention	11	22.50	26.373	
Pantothenic acid	Control	10	7.40	5.444	0.4
	Intervention	11	8.76	6.618	
Vitamin K	Control	10	106.74	97.499	0.2
	Intervention	11	191.48	259.023	
Phosphorus	Control	10	1606.08	1093.803	0.4
	Intervention	11	2109.34	1453.846	
Magnesium	Control	10	380.27	274.785	0.7
	Intervention	11	461.91	298.362	
Zinc	Control	10	18.27	26.200	0.1
	Intervention	11	14.86	8.338	
Copper	Control	10	1.81	1.165	0.1
	Intervention	11	2.81	3.068	
Manganese	Control	10	3.35	2.216	0.05
	Intervention	11	3.51	1.361	
Selenium	Control	10	90.30	76.397	0.4
	Intervention	11	125.42	91.977	
Fluoride	Control	10	727.60	671.907	0.7
	Intervention	11	837.30	567.026	
Chromium	Control	9	.03	.020	0.2
	Intervention	11	.05	.078	
Molybdenum	Control	4	2.93	3.343	0.9
	Intervention	4	2.90	3.190	
Dietary fiber	Control	10	35.51	29.493	0.3
	Intervention	11	34.86	21.360	
Soluble fiber	Control	10	.52	1.012	0.01*
	Intervention	11	.14	.142	

Insoluble fiber	Control	10	3.33	5.762	0.07
	Intervention	11	1.34	2.158	
Crude fiber	Control	10	2.51	1.774	0.2
	Intervention	11	1.85	1.363	
Total sugar	Control	10	96.85	50.206	0.5
	Intervention	11	96.86	71.418	
Glucose	Control	10	17.23	18.386	0.2
	Intervention	11	10.89	10.046	
Galactose	Control	10	.81	.923	0.3
	Intervention	11	1.21	1.676	
Fructose	Control	10	18.42	19.614	0.3
	Intervention	11	13.66	11.845	
Sucrose	Control	10	22.50	18.777	0.7
	Intervention	11	24.93	31.602	
Lactose	Control	10	2.47	2.126	0.2
	Intervention	11	3.50	4.309	
Maltose	Control	10	.23	.188	0.09
	Intervention	11	.08	.098	
sugar alcohol	Control	10	.00	.000	0.04*
	Intervention	11	.30	1.005	
Tryptophan	Control	10	847.60	809.637	0.4
	Intervention	11	1131.53	1401.870	
Threonine	Control	10	3026.18	3216.522	0.5
	Intervention	11	3971.84	5051.638	
Isoleucine	Control	10	3244.18	3073.000	0.3
	Intervention	11	4713.47	6138.726	
Leucine	Control	10	5603.22	5851.354	0.5
	Intervention	11	7398.50	8809.482	
Lysine	Control	10	5594.73	6489.483	0.5
	Intervention	11	7423.97	9923.634	
Methionine	Control	10	1756.99	2105.217	0.5
	Intervention	11	2386.96	3184.656	
Cystine	Control	10	925.10	742.371	0.3
	Intervention	11	1319.47	1565.093	
PHE	Control	10	3548.66	2887.511	0.4
	Intervention	11	4618.42	5126.195	
Valine	Control	10	3860.94	3320.176	0.3
	Intervention	11	5290.73	6085.727	
Arginine	Control	10	4569.11	4709.669	0.4
	Intervention	11	6021.29	7504.469	
Histidine	Control	10	2085.88	2359.308	0.5
	Intervention	11	2747.61	3533.487	
Alanine	Control	10	3702.11	4156.223	0.4

	Intervention	11	4983.45	6648.952	
Aspartic acid	Control	10	9485.97	8035.352	0.5
	Intervention	11	11086.60	12865.479	
Glutamic acid	Control	10	13699.60	11527.765	0.5
	Intervention	11	17158.47	18052.648	
Glycine	Control	10	3174.65	3272.895	0.5
	Intervention	11	4753.96	6718.563	
Proline	Control	10	3777.32	3001.916	0.5
	Intervention	11	5239.86	5317.484	
Serine	Control	10	3214.62	2762.344	0.3
	Intervention	11	4147.82	4325.512	
Caffeine	Control	10	109.30	78.745	0.3
	Intervention	11	79.23	65.732	
